# Antipsychotics and chronic dystonia at a Botulinum Toxin clinic

**DOI:** 10.4102/sajpsychiatry.v30i0.2270

**Published:** 2024-09-16

**Authors:** Mahlatse Thosago, Laila Asmal

**Affiliations:** 1 Department of Psychiatry, Faculty of Medicine and Health Sciences, Stellenbosch University, Cape Town, South Africa

**Keywords:** dystonia, Botulinum toxin, antipsychotics, chronic dystonia, tardive dystonia, neuroleptics

## Abstract

**Background:**

Chronic dystonia, characterised by sustained muscle contractions and abnormal postures, poses clinical challenges, especially when associated with antipsychotic medication use.

**Aim:**

To delineate the demographic and clinical profiles of adults with dystonia and examine the association with antipsychotic medication.

**Setting:**

Botulinum Toxin Clinic at Tygerberg Hospital, Cape Town, South Africa.

**Methods:**

We conducted a retrospective cohort study of adult patients seen at the Botulinum Toxin Clinic between January 2018 and June 2022.

**Results:**

Of the 119 patients studied, those assessed with antipsychotic-induced dystonia (32.69%) presented at a younger age (*p* < 0.001), were more likely female (*p* = 0.04), received higher average dose of Botulinum toxin (*p* < 0.001), and incurred a higher estimated Botulinum toxin treatment cost (*p* = 0.01) compared to those with primary dystonia. Logistic regression identified age and Botulinum toxin dose as factors associated with psychotropic-related dystonia (*p* = 0.005 and *p* = 0.012, respectively).

**Conclusion:**

Clinical and demographic factors are associated with dystonia in adults taking antipsychotic medication. These patients generally manifested symptoms at an earlier age, had a higher male prevalence, and required prolonged treatment with Botulinum toxin, leading to increased costs. In those assessed with antipsychotic-induced dystonia, a comorbid diagnosis of a mood disorder was more common than that of a psychotic disorder.

**Contribution:**

By identifying the demographic and clinical profile of individuals with dystonia because of antipsychotic medication, this study provides a basis for preventative strategies and enhanced patient care.

## Introduction

Chronic dystonia is a complex movement disorder characterised by persistent and involuntary muscle contractions that result in abnormal and often repetitive twisting movements or sustained postures.^1^ Dystonia can manifest in various body regions, affecting the neck, face, vocal cords, eyelids, and limbs, thus posing a significant healthcare challenge that spans the age spectrum, impacting both adults and children.^2,3^

The classification of dystonia serves as a cornerstone of its clinical evaluation and involves two primary axes: Axis I, encompassing clinical descriptors, and Axis II, focused on aetiological factors.^4^ Axis I enables healthcare professionals to visually categorise dystonia presentations, aiding in treatment decisions. It includes subtypes such as generalised dystonia, segmental dystonia, multifocal dystonia, focal dystonia, and hemidystonia, which provide insights into the extent and anatomical distribution of muscle contractions.^1,5^ Additionally, clinical descriptions consider the age of onset and temporal aspects, aiding in prognosis and understanding the variability of symptom manifestation.^4^

There appears to be a bidirectional relationship between dystonia and mental health, with depression and anxiety being the most common mental illnesses associated with dystonia.^6^ Reported rates of depression among people with dystonia range from 19% to 47%, and anxiety disorders from 25% to 69%.^6^ A study by Gundel et al.^7^ demonstrated that individuals with cervical dystonia experience rates of depression that are double, rates of anxiety that are six times higher, and rates of social phobia that are 10 times higher than those in the general population. Additionally, there are elevated rates of depression and anxiety in patients with dystonia even prior to the onset of the movement disorder. Carriers of the DYT1 gene mutation, which increases the risk of dystonia, exhibit higher and earlier onset rates of major depressive disorder compared to non-carriers.^8^

These comorbidities contribute to the substantial impact of dystonia on individuals’ lives. Moreover, research indicates that people with cervical dystonia and blepharospasm may experience sleep problems and pain, further emphasising the intricate relationship between dystonia and mental health.^9,10,11^

Antipsychotic medications can induce dystonia, leading to a condition known as Tardive Dystonia (TD), which is a form of chronic dystonia.^12^ The pathophysiological mechanisms of TD, such as aberrant dopaminergic activity within the nigrostriatal tract, remain complex and not fully understood.^13^ Approximately 1% – 2% of individuals taking long-term antipsychotic treatment develop TD, making it a notable extrapyramidal side effect.^13^ Risk factors for TD include younger age, male sex, previous history of dystonic reactions and stimulant use.^14^

The primary aim of this study was to examine the clinical characteristics and prevalence of chronic dystonia among all adults attending the Botulinum Toxin Clinic (BTC) at Tygerberg Hospital, with a specific focus on associations with antipsychotic medication use. This includes comparing demographic and clinical profiles of patients with antipsychotic-associated dystonia to those with other forms of dystonia. The BTC at Tygerberg Hospital serves as a specialised centre for administering Botulinum toxin, a first-line treatment for chronic dystonia. The clinic was chosen for this study because of its role in treating a broad spectrum of dystonia cases, including those induced by antipsychotic medications. Its experience with various types of dystonia, along with access to a diverse patient population, provided an important perspective on the presentation and clinical features of antipsychotic-associated dystonia which allowed a comparison to other forms of chronic dystonia within a clinical setting.

## Research methods and design

### Study design

This retrospective cohort study explored the clinical and demographic characteristics of adult patients with chronic dystonia attending a BTC at Tygerberg Hospital between January 2018 and June 2022.

### Study setting

The study was conducted at the BTC of the Movement Disorders Unit of the Department of Neurology at Tygerberg Hospital. Patients with chronic dystonia initially undergo assessment at a general neurology clinic where neurologists determine the aetiology and appropriate management of the dystonia. In cases where dystonia is assessed as induced by antipsychotic medications, neurologists collaborate with psychiatrists to review and adjust the type and dosage of these medications. If Botulinum toxin treatment is indicated, patients are referred to the BTC. The BTC administers Botulinum toxin injections, and coordinates additional treatments such as physiotherapy, and medications such as clonazepam and baclofen as needed.

Tygerberg Hospital is located in the Eastern Metropole of Cape Town and is the largest public tertiary hospital in the province. Patients are mostly referred to the hospital from primary and secondary healthcare facilities situated in the drainage area of the hospital, which are predominantly low-income communities and rural areas.

### Study sample

The study population consisted of all adult (aged 18 and above) patients seen at the Tygerberg Hospital BTC for evaluation and management of their dystonia. Inclusion criteria were adults aged 18 years or older who were evaluated and managed at the BTC between 01 January 2018 and 30 June 2022, and had a confirmed diagnosis of dystonia which included a clinically assessed aetiology. Patients were excluded if the aetiology of their dystonia was unclear (specifically whether it was antipsychotic-induced or not) or were under 18 years of age at the time of their BTC consultation.

The BTC diary, clinical notes from Enterprise Content Management (ECM) and the physical clinical folders were used to determine the number of patients accessing the BTC in the specified period. Of the *n* = 149 case files reviewed, *n* = 119 met the inclusion criteria and were suitable for further analysis.

### Data collection

The BTC runs on the first Friday of every month and the Neurology Department keeps a written database of all patients seen. All patients at the clinic routinely had an intake assessment where demographic and clinical details were captured. The medical records of these patients were reviewed to obtain the demographic (sex, age, referral hospital) and clinical characteristics (primary vs. secondary, age of onset, anatomical distribution, relationship to antipsychotic medication, current psychiatric medication) of each patient using a standardised data-collection form. All patients included in the study were confirmed to have dystonia by a neurologist through the findings from the history taking, clinical examination, and electromyography studies (EMG studies). The relationship between dystonia and antipsychotic medication was determined through a detailed review of the BTC notes including the type, dosage, and duration of antipsychotic medication use, as well as the timing of dystonia symptom onset relative to the initiation of antipsychotic treatment. To ensure accuracy, we cross-referenced the neurologists’ diagnoses and medication details with electronically available pharmaceutical prescriptions for all patients.

### Data analysis

The data were analysed using Stata version 17 computer program (College Station, TX, Stata Corporation; 2021). Numerical variables were reported as either mean and standard deviation (s.d.) or median and interquartile range (IQR) based on their distribution. Categorical variables were summarised as absolute counts and percentages. The Chi-square test was used to determine statistical differences between categorical variables and the T-test or Mann–Whitney tests were used for continuous variables. Logistic regression analysis was used to identify variables associated with secondary dystonia because of antipsychotic medication, controlling for potential confounding variables. A *p*-value < 0.05 was used to determine statistical significance in hypothesis testing. Odds ratios (ORs) and 95% confidence intervals (95% CIs) were derived from the logistic regression analysis.

### Ethical considerations

Ethical approval was obtained from the Stellenbosch University, Health Research Ethics Committee (No. S22/10/208) and a waiver of informed consent was granted. The study was conducted in accordance with the South African Clinical Good Practice Guidelines^15^ and the Declaration of Helsinki.^16^ All data were anonymised to ensure privacy and confidentiality of participants’ personal information, with each participant assigned a unique identifier.

## Results

### Clinical and demographic characteristics

Of the 119 participants, the average age at presentation was 53.46 years, with females (63%) outnumbering males (Table 1). The most common form of dystonia was focal (36.13%), and the majority (67.23%) had a static disease course. Concerning aetiology, acquired causes accounted for 43.7% (*n* = 52), of which 32.69% (*n* = 17) were because of antipsychotic medication, and other psychotropic medication (3.85%, *n* = 2) with most (63.16%, *n* = 12) developing the condition after the first 5 years of medication onset. A positive psychiatric history was found in 26.05% (*n* = 31) of the sample and within this group, mood disorders (48.38%, *n* = 15) and psychotic disorders (45.16%, *n* = 14) were the predominant diagnoses.

**TABLE 1:** Demographic and clinical characteristics of the total sample presenting with dystonia (*N* = 119).

Variable	Mean	s.d.	*n*	%
Age at first presentation of dystonia (years)	53.46	13.9	-	-
**Sex**				
Female	-	-	75	63.0
Male	-	-	44	37.0
**Body distribution of dystonia**				
Generalised	-	-	18	15.1
Multifocal	-	-	4	3.4
Hemidystonia	-	-	24	20.2
Segmental	-	-	30	25.2
Focal	-	-	43	36.1
**Disease course of dystonia**				
Progressive	-	-	39	32.8
Static	-	-	80	67.2
**Aetiology of dystonia**				
Acquired	-	-	52	43.7
Idiopathic	-	-	60	50.4
Inherited	-	-	7	5.9
**Acquired dystonia**				
Antipsychotic medication	-	-	17	32.7
Other psychotropic medication†	-	-	2	3.8
Parkinson’s disease	-	-	18	34.6
Other	-	-	15	28.9
Positive psychiatric history (yes)			31	26.1
**Psychiatric diagnosis**				
Psychotic disorder	-	-	14	45.2
Mood disorder	-	-	15	48.8
Anxiety disorder	-	-	2	6.5
**Time to dystonia after psychotropic onset**				
In first 5 years of psychotropic onset	-	-	5	26.3
After first 5 years of psychotropic onset	-	-	12	63.2
Unknown	-	-	2	10.5

s.d., standard deviation.

† , *n* = 1: duloxetine, quetiapine, and lamotrigine.

Table 2 contrasts the demographic and clinical characteristics of patients with psychotropic-related dystonia with other dystonias. Those in the psychotropic group were younger at first presentation (mean age of 43.53 vs. 55.35 years, *p* < 0.001) and had fewer females (42% vs. 67%, *p* = 0.04). While the body distribution patterns varied, differences were not significant (*p* = 0.08). The psychotropic group received a higher mean Botulinum toxin dose (*p* < 0.001) and had increased estimated lifetime costs in South African Rands (*p* = 0.01).

**TABLE 2:** Demographic and clinical characteristics in psychotropic related dystonia versus other forms of dystonia.

Variable	Psychotropic dystonia (*N* = 19)						Other (*N* = 100)						*p*
	Mean	s.d.	*n*	%	Median	IQR	Mean	s.d.	*n*	%	Median	IQR	
Age at first presentation in years	43.53	14.73	-	-	-	-	55.35	13.09	-	-	-	-	**< 0.001**
Female sex	-	-	8	42.1	-	-	-	-	67	67.0	-	-	**0.040**
**Body distribution of dystonia**													
Focal	-	-	3	15.8	-	-	-	-	40	40.0	-	-	-
Generalised	-	-	5	26.3	-	-	-	-	13	13.0	-	-	-
Hemidystonia	-	-	2	10.5	-	-	-	-	22	22.0	-	-	-
Multifocal	-	-	1	5.3	-	-	-	-	3	3.0	-	-	-
Segmental	-	-	8	42.1	-	-	-	-	22	22.0	-	-	-
Botulinum toxin dose	126.58	71.38	-	-	-	-	74.75	65.88	-	-	-	-	**< 0.001**
**Disease course of dystonia**													
Progressive	-	-	8	42.1	-	-	-	-	31	31.0	-	-	-
Static	-	-	11	57.9	-	-	-	-	69	69.0	-	-	-
Estimated lifetime Botulinum Cost, in ZAR	-	-	-	-	10 120	1 265 to 17 710	-	-	-	-	6 325	1 265 to 20 240	**0.010**

s.d., standard deviation; IQR, interquartile range; ZAR, South African Rands.

Note: Bold *p*-values denote significance at *p* < 0.05.

### Logistic regression analysis

Logistic regression analysis of the association between psychotropic-related dystonia and clinical and demographic characteristics is presented in Table 3. The odds of an individual having psychotropic-related dystonia dropped by 6% with each additional year of age at their first presentation (OR: 0.94, 95% CI: 0.91 to 0.98, *p* = 0.005). Additionally, higher doses of Botulinum Toxin were observed in the psychotropic-related dystonia group compared to the non-psychotropic group (OR: 1.009, 95% CI: 1.00 to 1.02, *p* = 0.012).

**TABLE 3:** Logistic regression analysis comparing socio-clinical factors associated with psychotropic-related dystonia versus other forms of dystonia.

Variable	OR	95% CI	*p*
Age at first presentation (years)	0.94	0.91 to 0.98	**0.005**
**Sex**			
Female	2.21	0.74 to 6.61	0.154
Male	Reference	-	-
Botox dose	1.009	1.00 to 1.02	**0.012**

OR, odds ratio; 95% CI, 95% confidence interval.

Note: Bold *p*-values denote significance at *p* < 0.05.

## Discussion

In this study, we aimed to investigate the association between antipsychotic medication and the clinical features of chronic dystonia among adults attending a BTC at a tertiary state facility in Cape Town, South Africa. A key finding was the notable use of psychotropic medications, especially antipsychotic drugs, in the clinic’s dystonia cases.

Strikingly, among those with a documented psychiatric history, a primary diagnosis of a mood disorder was more common than that of a psychotic disorder. Dystonia patients taking psychotropic medications generally had a manifestation of symptoms at an earlier age, had a higher male prevalence, and required higher doses of Botulinum Toxin, leading to increased costs.

Our observations are consistent with established literature in terms of dystonia’s clinical presentation and its varied anatomical distribution.^1,2^ We further identified distinct demographic differences between antipsychotic-induced chronic dystonia patients and their primary or other secondary dystonia counterparts. Earlier research by Kiriakakis et al.^17^ and Keepers and Casey^14^ has highlighted similar demographic risk factors. Our study underscores the need for careful monitoring of dystonic symptoms in younger patients on antipsychotics and suggests possible sex-specific susceptibility factors.

Dystonia’s classification involves a dual-pronged approach encompassing clinical manifestation (Axis I) and aetiology (Axis II). Axis I primarily focuses on the observable symptoms of dystonia, providing an immediate clinical framework.^5,18^ In contrast, Axis II outlines the disorder’s biological origins, involving detailed neurobiological and genetic evaluations.^4^ Because of constraints like limited investigative resources even at tertiary, academic hospitals such as Tygerberg Hospital, the dichotomous system of primary and secondary classification is more feasible. The management strategy often remains consistent regardless of a deeper Axis II approach.

Pharmacological interventions, notably Botulinum toxin injections, are vital for dystonia management. However, treatment access is a challenge, especially at South African state facilities where Botulinum Toxin Type A is restricted because of cost (R1265 per 100-unit vial). However, despite the lifelong treatment required for conditions like TD, our study estimates the median lifetime costs to be relatively modest, at R10 120. Given the young age of onset in people with psychiatric disorders, and the pronounced physical and psychological impact of the condition,^10,11^ it underscores the need for broader access to Botulinum toxin and thoughtful healthcare resource allocation.

The study is one of the first in Africa to compare TD among patients treated with Botulinum Toxin for psychotropic-related and other forms of chronic dystonia. The study’s findings have practical implications for the early identification and management of antipsychotic-induced dystonia, potentially improving patient outcomes.^13,19^ However, there are a few potential limitations that need to be highlighted. Firstly, it was a retrospective study, which is subject to inherent limitations, such as reliance on historical medical records and potential recall bias. Secondly, the study’s findings may have limited generalisability beyond the specific healthcare context of Tygerberg Hospital which may affect the applicability of findings to broader populations. While the sample size is substantial compared to similar studies, larger cohorts may provide more robust insights, particularly for sub-analyses which should be the focus of future studies. This would include further exploring the relationship between TD, mood disorders, and antipsychotic medication including class and dose of antipsychotic, and whether males who develop TD were more likely than females to be treated with antipsychotic medication for mood disorders as first line or adjunctive medication.

## Conclusion

In conclusion, these findings underscore the importance of close monitoring for dystonia signs in antipsychotic medication users, emphasising cautious prescription. Further research, ideally prospective, with a larger sample size and multicentre is needed to corroborate these findings and explore underlying mechanisms driving these differences in presentation.
